# Supplementary material for: Comparison of potential drinking water source contamination across one hundred U.S. cities
Source: Nat Commun. 2021 Dec 13;12:7254. doi: 10.1038/s41467-021-27509-9 (PMC8669013; doi:10.1038/s41467-021-27509-9)
Supplement: Supplementary file 1 — Supplementary Information [file 41467_2021_27509_MOESM1_ESM.pdf]

Supplementary materials for Turner et al. "Anthropogenic activities in source watersheds lead to wide disparities in potential contamination of urban drinking water supplies in the United States"

Contents:

Supplemental Figure 1 – Point and nonpoint PPCS metrics for all cities, with and without groundwater included.

Supplemental Table 1 – Details of all relevant health-based water supply violations affecting major US cities.

Supplemental Figure 2 (a - d) – E90 Effluent violations.

Supplemental Figure 3 – Worked example of Indirect Potable Reuse implementation.

Supplemental Figure 1

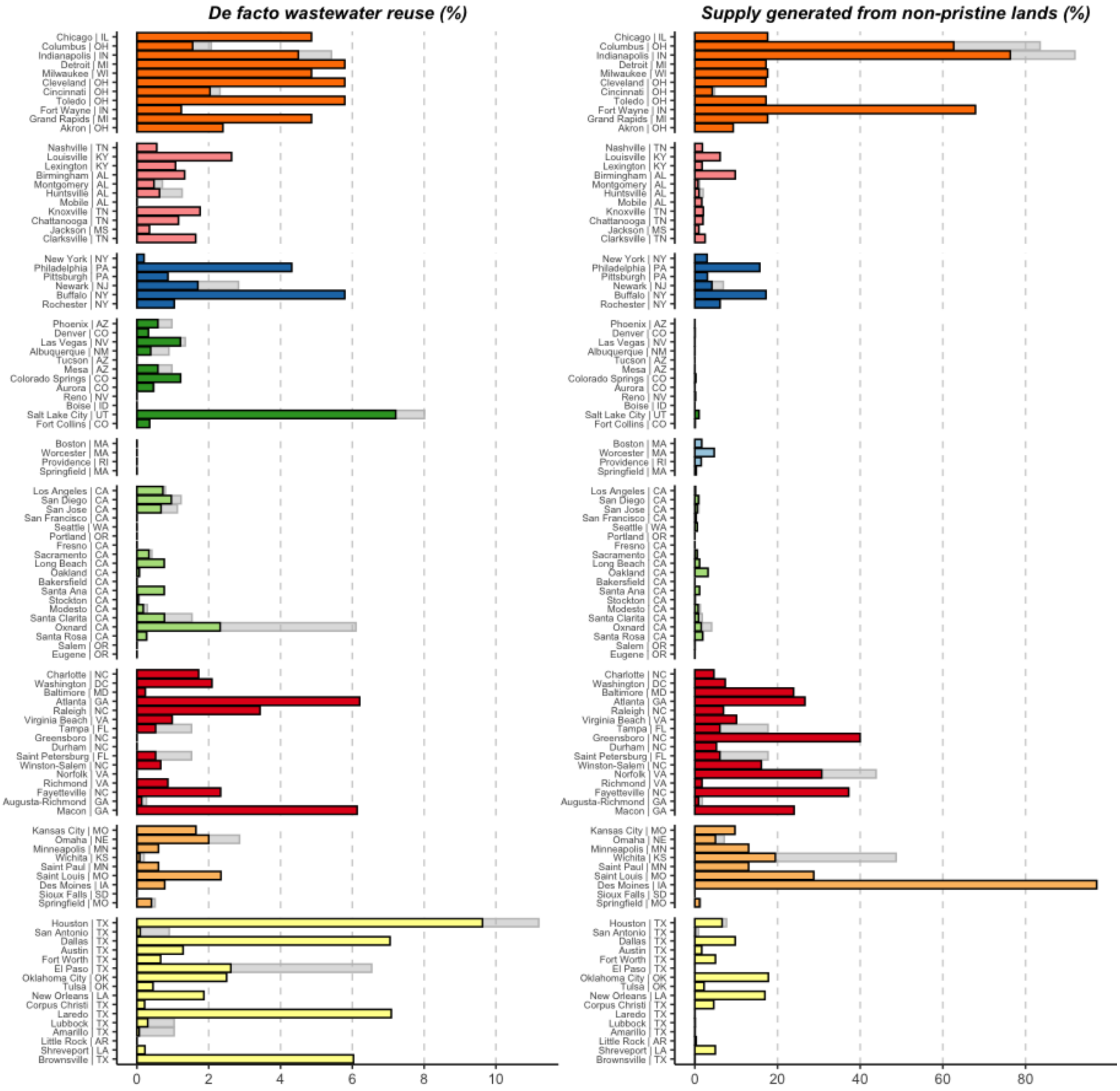

De facto wastewater reuse (i.e. point PPCS) and supply generated on non-pristine lands (i.e., nonpoint PPCS) for all cities studied. Results show all supply sources combined (i.e., including groundwater contribution) and surface water sources only (grey extensions to each bar).

Supporting Table 1. Water supply violations summary.

Water supply violations for community water systems are recorded in EPA Safe Drinking Water Information System (SDWIS) Violations Report. We obtained Public Water System (PWS) IDs for all 116 cities in this study. We filtered the SDWIS Violations Report for these PWS IDs, and for “health-based” violations (i.e., monitoring, reporting, and other violations removed) for the following rules: Inorganic Chemicals (IOCs), Synthetic Organic Chemicals (SOCs), Nitrates, Arsenic, and Radionuclides. This removes violations unrelated to human activity in watersheds (e.g., violations related to disinfection processes or plumbing). A “health-based violation” means an amount of contaminant exceeded the safety standard.

City	PWS ID	PWS Name	# violations	Rule(s)	Year(s)
Columbus, Ohio	OH2504412	Columbus Public Water System	5	Nitrates	2006, 2007, 2015, 2016
Louisville, Kentucky	KY0560258	Louisville Water Company	3	IOCs	2001
Des Moines, Iowa	IA7727031	Des Moines Water Works	1	Nitrates	1984
Fort Wayne, Indiana	IN5202020	Fort Wayne – 3 Rivers Filtration Plant	1	SOCs	1994
Fresno, California	CA1010007	City of Fresno	2	Nitrates, SOCs	1998, 2010
Modesto, California	CA5010010	City of Modesto	3	Nitrates, Radionuclides	1998, 2008, 2013
Santa Clarita, California	CA1910017	Santa Clarita Valley Water Agency	2	Nitrates	2003, 2007
Tucson, Arizona	AZ0410112	City of Tucson	1	Nitrates	1981
Mesa, Arizona	AZ0407095	City of Mesa	3	Nitrates, IOCs	1981, 1982, 2006

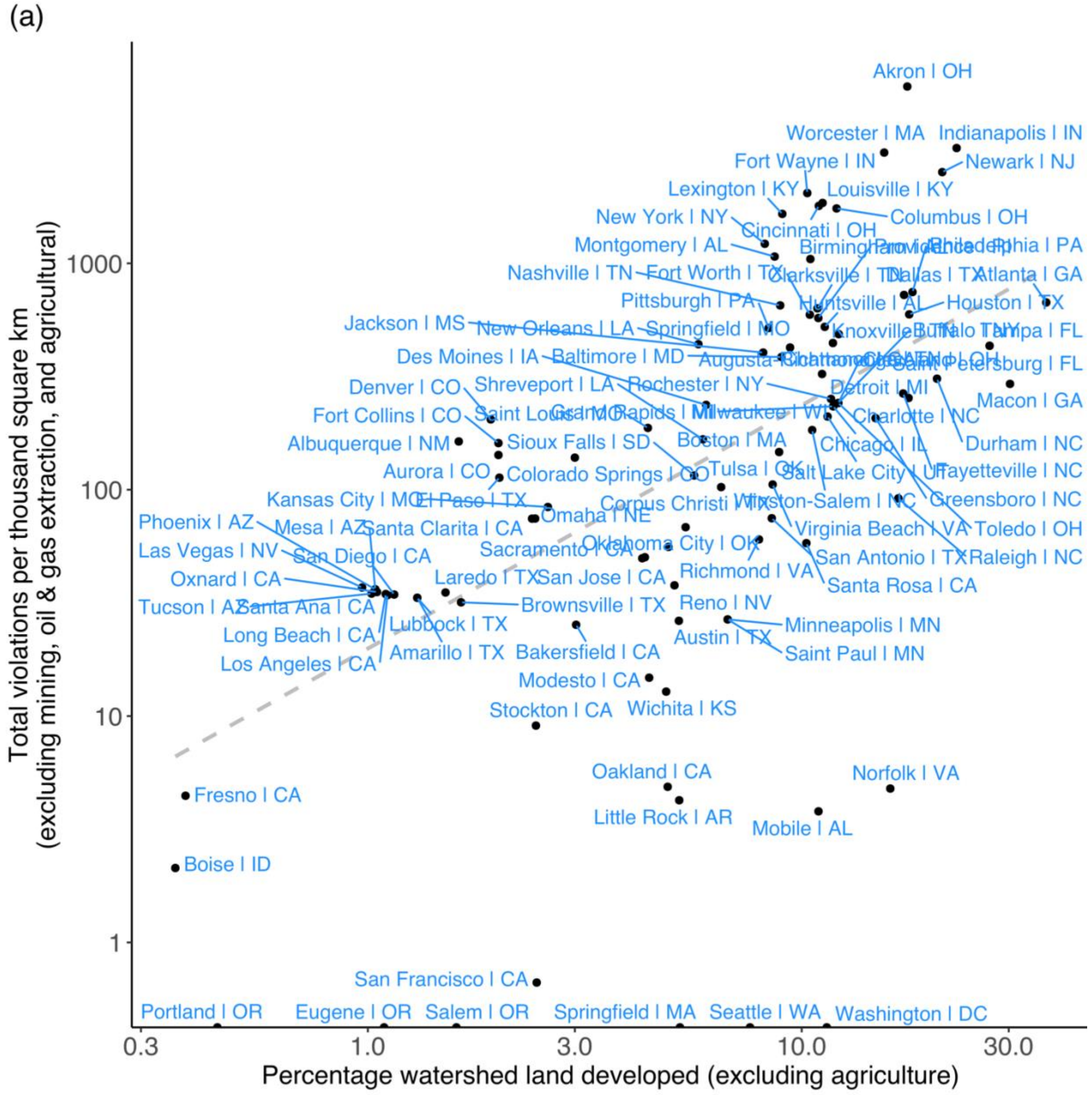

Supp. Fig 2a. Watershed area developed for non-agricultural purposes (%) versus E90 Effluent Violations (all categories except mining, gas extraction, and agriculture).

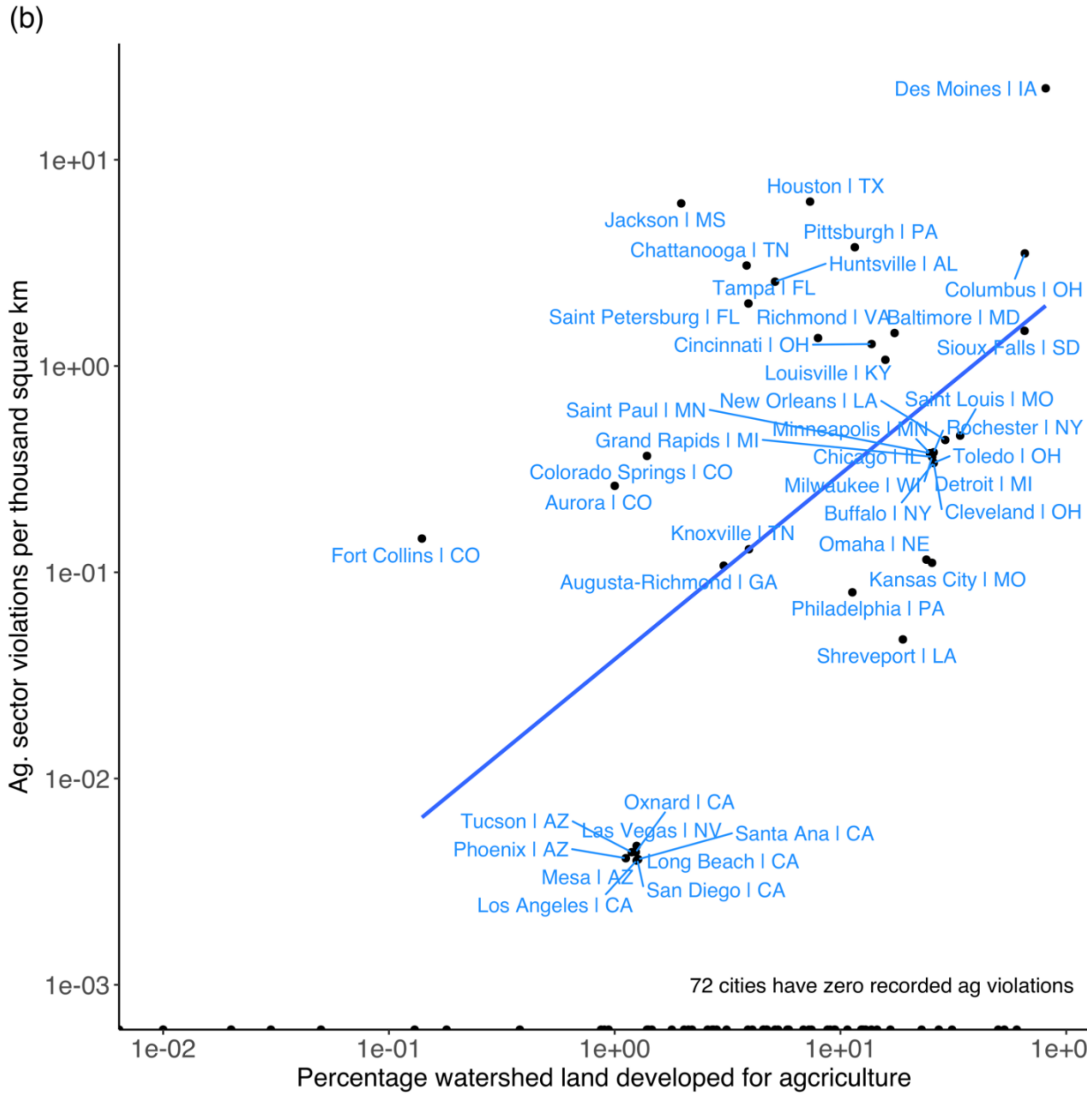

Supp. Fig 2b. Watershed area developed for non-agricultural purposes (%) versus E90 Effluent Violations (all categories except mining, gas extraction, and agriculture).

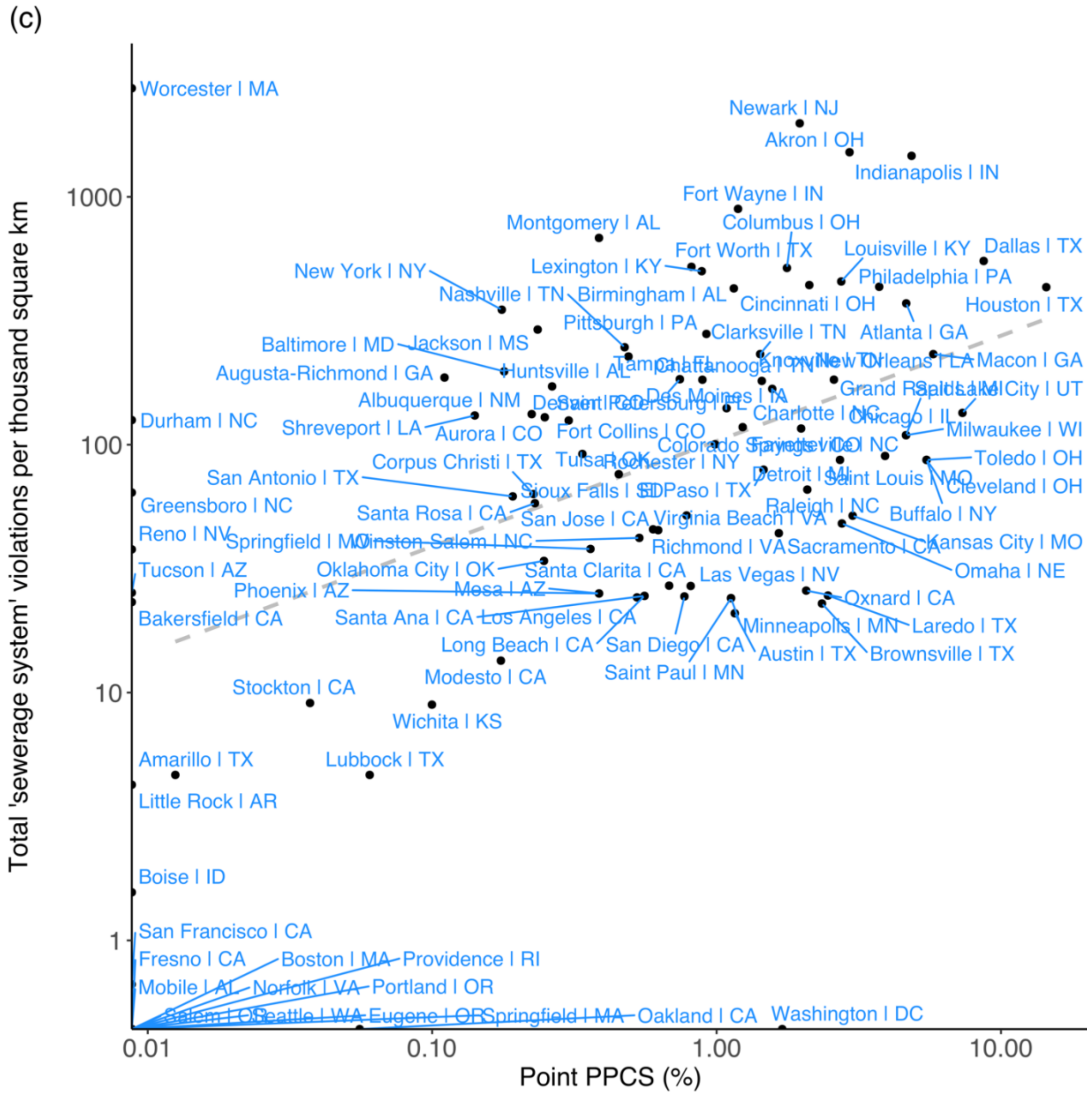

Supp. Fig 2c. Point PPCS versus wastewater effluent violations.

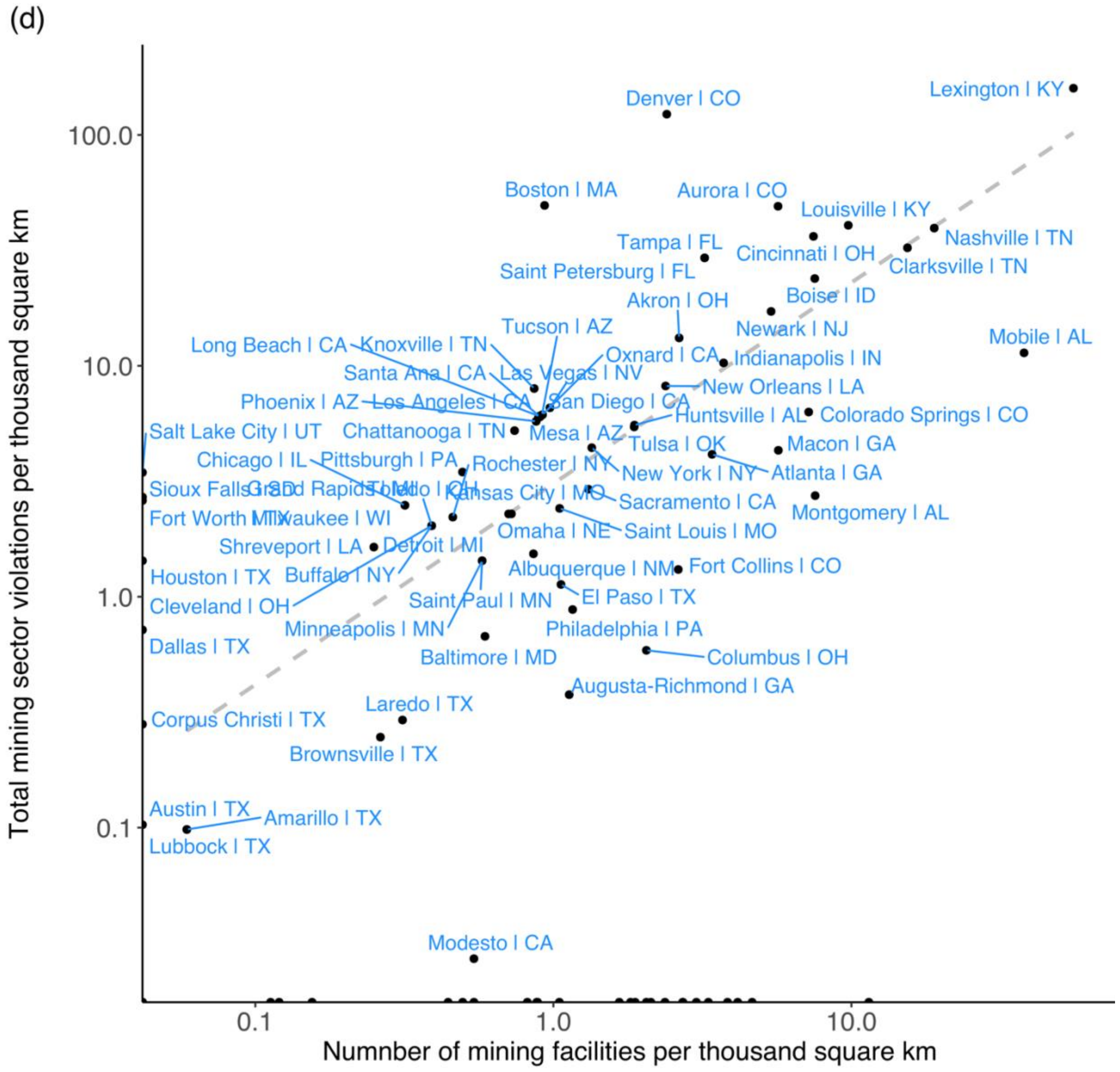

Supp. Fig 2d. Mining facilities versus mining facility violations.

Supplemental Figure 3

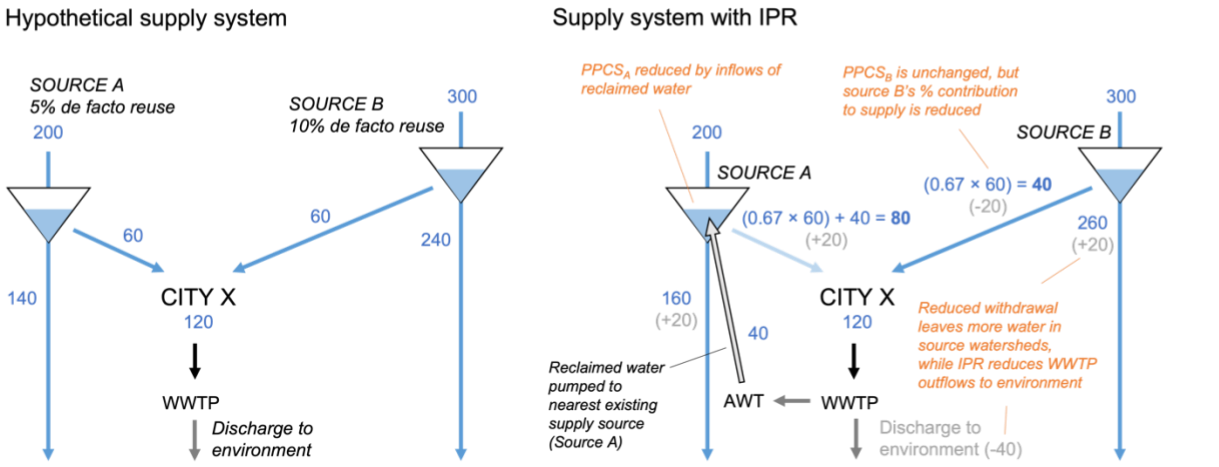

Point PPCS calculation:
$(PPCS_A \times contribution_A + PPCS_B \times contribution_B)$ / demand
= (0.05 × 60 + 0.1 × 60) / 120
= (3 + 6) / 120 = 0.075
= **7.5 %**

Point PPCS calculation:
$([PPCS_A \times inflow_A / (inflow_A + inflow_{AWT})] \times contribution_A$
$+ PPCS_B \times contribution_B)$ / demand
= ((0.05 × 200) / 240 × 80 + 0.1 × 40) / 120 = (0.04 × 80 + 0.1 × 40) / 120 = 0.061
= **~6.1 %**

Reclaimed water calculation:
$(inflow_{AWT} / inflow_{A\_TOTAL} \times contribution_A)$ / demand
= (40 / 240 × 80) / 120 = 0.011
= **~11.1 %**

Point PPCS calculation (if reclaimed water is considered potentially contaminated)
Point PPCS exc. reclaimed water + reclaimed water
= 6.1 + 11.1
= **17.2 %**

Example of IPR implementation and PPCS calculations for a hypothetical system with two water supply sources (Source A, with 5% de facto reuse and Source B, with 10% de facto reuse). With IPR, "city X" returns water commensurate with 1/3 of total water supply back to the nearest supply source. Withdrawals from each natural water sources are reduced by 1/3 to accommodate this new supply.
